# Management of Malignant Bowel Obstruction in Patients with Gynaecological Cancer: A Systematic Review

**DOI:** 10.3390/jcm13144213

**Published:** 2024-07-19

**Authors:** Richárd Tóth, Zsófia Tóth, Lotti Lőczi, Marianna Török, Nándor Ács, Szabolcs Várbíró, Márton Keszthelyi, Balázs Lintner

**Affiliations:** 1 Department of Obstetrics and Gynaecology, Semmelweis University, 1082 Budapest, Hungary; toth.richard@semmelweis.hu (R.T.); toth.zsofia99@stud.semmelweis.hu (Z.T.); keszthelyi.lotti.lucia@semmelweis.hu (L.L.); torok.marianna@semmelweis.hu (M.T.); acs.nandor@semmelweis.hu (N.Á.); varbiro.szabolcs@semmelweis.hu (S.V.); lintner.balazs.zoltan@semmelweis.hu (B.L.); 2 Workgroup of Research Management, Doctoral School, Semmelweis University, 1085 Budapest, Hungary; 3 Department of Obstetrics and Gynaecology, University of Szeged, 6725 Szeged, Hungary

**Keywords:** malignant bowel obstruction (MBO), gynaecologic cancer, surgical management, non-surgical treatment, palliative care

## Abstract

**Objectives:** This systematic review aimed to evaluate current surgical and non-surgical management strategies for malignant bowel obstruction (MBO) in patients with gynaecological cancer. **Methods:** Comprehensive literature searches were conducted across MEDLINE, Embase, CENTRAL, and Scopus, without restrictions on language or publication date. Following the removal of duplicates, 4866 articles were screened, with 34 meeting the inclusion criteria. **Results:** Surgical intervention remains the definitive treatment for MBO, offering longer symptom-free periods and improved survival, particularly when conservative methods fail. However, the selection of surgical candidates is crucial due to the high risk of morbidity and the potential for significant complications. Non-surgical treatments, such as the use of Gastrografin, Octreotide, and Dexamethasone, along with invasive procedures like nasogastric tubing, percutaneous gastrostomy, and stent placement, offer varying degrees of symptom relief and are often considered when surgery is not feasible. **Conclusions:** In this article we provide a potential therapeutic algorithm for the management of patients with MBO. This review underscores the urgent need for high-quality research to develop clear, evidence-based guidelines for MBO management in patients with gynaecologic cancer. Establishing standardised protocols will improve patient outcomes by aiding clinicians in making informed, individualised treatment decisions.

## 1. Introduction

Gynaecological malignancies were responsible for 680,000 deaths globally in 2022, with incidence rates continuing to rise. According to WHO calculations, mortality from these types of cancers is predicted to reach 1.1 million cases per year by 2040 [1]. Although various preventive measures and advancements in targeted therapies [2] have extended survival rates, clinicians are frequently confronted with complex decisions regarding palliative care. While administering antitumor therapy or performing surgeries are guided by well-established protocols, palliative care decisions often rely on individualised clinical judgement due to the lack of robust evidence [3].

A particular area of focus is on the management of malignant bowel obstructions (MBOs). Malignant bowel obstruction is a clinical syndrome that is caused by malignant disease, antitumoral treatment, or its complications, and once it occurs it tends to recur. For this reason, some authors entitle it an occlusive state rather than a single event [4]. 

Studies indicate that MBO occurs in 25–60% of patients with gynaecological cancers [5,6]. The consequences of occlusion evoke severe symptoms, significantly damage the quality of life of the patients, and can be life-threatening.

### 1.1. Pathomechanism of Malignant Bowel Obstruction

Bowel obstructions can be categorised into mechanical and functional types, each requiring different management strategies. Mechanical obstructions, which include intraluminal causes like faecal impaction or intraluminal tumour growth and extraluminal causes like tumours or adhesions, physically block the intestines. Functional obstructions, such as paralytic ileus or pseudo-obstruction, involve impaired intestinal motility without a physical blockage. Additionally, obstructions can be partial or complete and may occur in the small or large bowel, with each presenting distinct symptoms and clinical challenges. Accurate differentiation of these types is crucial for effective treatment and improving patient outcomes.

Bowel occlusions typically develop gradually, though symptoms can appear suddenly, and the causes are often multifactorial [7]. 

Mechanically, bowel occlusion can result from infiltration of the bowel or mesentery by a tumour or from a bulky tumour exerting external pressure on the bowel [8]. In colorectal cancer, the primary cause of occlusion is intraluminal tumour growth [9]. Conversely, intraluminal occlusion is less common in gynaecologic tumours.

**Figure 1:**
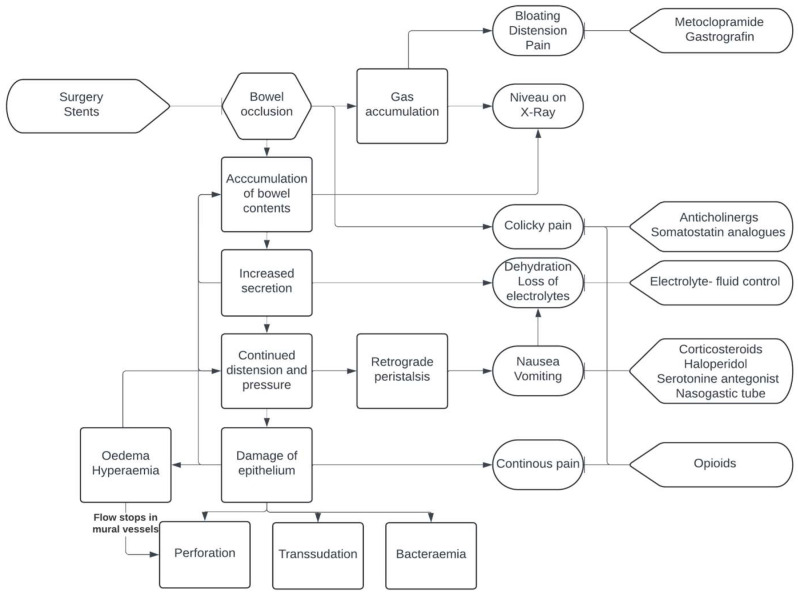
Pathogenesis of and points of intervention in MBO.

Functional bowel obstruction, such as adynamic ileus, represents another mechanism of transit disorder contributing to bowel occlusion [10]. These disorders can arise from infiltration of the muscular layer, nerves of the bowel, or the celiac plexus, leading to decreased motility. These can be exacerbated by the side effects of chemotherapy or pain medications. Although it is rare in gynaecologic cancer patients, paraneoplastic syndrome can also contribute to functional obstructions. Furthermore, intra-abdominal adhesions, which may form after surgery, chemotherapy, or radiotherapy, play a significant role in the development of bowel occlusions [11]. A summary of MBO pathogenesis is presented in Figure 1.

### 1.2. Diagnosis of Malignant Bowel Obstruction

The diagnosis of MBO is established by clinical symptoms and a history of malignant disease. Nausea and vomiting, abdominal pain and distension, and constipation are the leading signs. In 10–20% of patients bowel obstruction is the first presentation of malignant disease, which is associated with worse prognosis [12,13]. 

Besides clinical symptoms and physical examination, X-ray or CT are the cornerstones of the diagnosis. Intraluminal fluid levels, preocclusive distension, and postocclusive normal bowel diameter are diagnostic for this disease [14]. 

### 1.3. Management of Malignant Bowel Obstructions (MBOs)

In the management of MBOs, a variety of treatment options are available. In the diagnostic process, diatrizoate meglumine plays an important role, but at the same time this can be therapeutic and potentially speed recovery as well [15]. Possessing high osmotic activity and a mild laxative effect, diatrizoate meglumine draws fluid to the bowel lumen, reducing wall oedema and stimulating peristalsis [16]. In cases of non-responders to diatrizoate meglumine, the next therapeutic step is the application of conservative methods, usually a combination of opioids, corticosteroids, and anti-secretory drugs [17]. 

As the main pathogenic event is the accumulation of bowel content, therapeutic efforts are made towards its reduction. Somatostatin analogues (octreotide, lanreotide) and anticholinergics such as hyoscine butylbromide decrease bowel motility and bowel and pancreatic secretion [18]. 

Pain in the majority of the cases is severe and colicky; its alleviation is hardly possible with the exclusive use of non-steroids. Thus, opioids remain commonly used due to their double effect: analgesia and decreased bowel motility.

Corticosteroids having anti-inflammatory and anti-secretory attributes, and can effectively reduce the amount of intraluminal content and wall oedema by promoting water and salt absorption [19]. 

Placement of a nasogastric tube is a beneficial temporary intervention, as it effectively decompresses the stomach, relieving symptoms such as abdominal distension, pain, and nausea caused by fluid accumulation. This decompression reduces the risk of aspiration, which can have a high mortality rate if vomiting occurs. Additionally, nasogastric tubes can provide a route for medication and nutrition in patients unable to tolerate oral intake, ensuring continued support during treatment [20]. 

Percutaneous endoscopic gastrostomy (PEG), a procedure where a feeding tube is placed directly into the stomach through the abdominal wall, is often used for patients with gastrointestinal obstructions. PEG is indicated for long-term enteral nutrition in conditions like distal gastric obstruction, which prevents adequate oral intake. It is also used for gastric decompression, helping to relieve symptoms and prevent complications associated with obstructions in the gastrointestinal tract [21]. Stent placement in the obstructed bowel is a minimally invasive procedure typically performed endoscopically to relieve blockage and restore function, providing immediate symptom relief and improving quality of life. Though effective, it can have complications such as stent migration, perforation, and re-obstruction, necessitating careful patient selection and expert execution. Overall, stent placement plays a vital role in the palliative management of MBO, offering a balance of efficacy and safety for patients with advanced malignancies [22].

Surgical interventions in these cases might be challenging for the surgeon and demanding for the patient. An eye must be kept on the intention of the treatment, as the goal is not cytoreduction and resolution of all of the occlusions. Types of operations vary from bowel resection to bypass or stoma formation, according to the localisation and possible multiplicity of the obstruction sites.

Among patients with gynaecological cancer, management of MBO presents a significant clinical challenge due to the lack of clear guidelines and standardised protocols. This ambiguity complicates decision-making processes for clinicians aiming to improve quality of life for affected patients. Surgical interventions, while associated with higher morbidity and mortality rates, often provide the most definitive solution by offering prolonged symptom-free periods and potentially more effective relief from obstruction [5]. Conversely, conservative treatments tend to have lower morbidity but do not significantly extend survival [23,24]. This necessitates a careful, individualised approach to patient care. Recently, the Multinational Association for Supportive Care in Cancer (MASCC) highlighted the need for a multidisciplinary approach to manage malignant bowel obstruction (MBO) in patients with cancer and to support their families [17]. Our review, based on a robust literature search, builds on these recommendations by systematically evaluating current surgical and non-surgical strategies for MBO in patients with gynaecological cancer. By synthesising the latest data, we aim to enhance clinical decision-making and improve patient outcomes, addressing critical gaps in the existing literature and underscoring the importance of prospective, innovative studies to assist clinicians navigating this complex and uncertain field.

## 2. Materials and Methods

The study’s protocol was registered on PROSPERO under reference number CRD42024543407. We declare that no Preferred Reporting Items for Systematic Reviews and Meta-Analyses (PRISMA) registration was performed for this study; however, our systematic review adheres to the guidelines outlined in the PRISMA 2020 Statement. Furthermore, we adhered to the recommendations provided in Version 6.3 of the Cochrane Handbook for Systematic Reviews of Interventions.

### 2.1. Eligibility Criteria

Our analysis encompassed research involving patients diagnosed with gynaecologic cancer experiencing malignant bowel obstruction, identified through clinical symptoms or radiological examination.

### 2.2. Information Sources

We conducted a systematic literature search in four medical databases––MEDLINE (via PubMed), Embase, CENTRAL, and Scopus––from inception to 10 May 2024.

### 2.3. Search Strategy

We applied the following search key for all fields in the given search engines: (gynaecological cancer OR gynaecologic oncology OR gynaecological tumour) AND (malignant bowel obstruction OR MBO OR intestinal obstruction OR malignant gastrointestinal obstruction). No language or other restrictions were imposed.

### 2.4. Selection Process

Following a systematic search of the databases and subsequent duplicate removal, selection was conducted according to PICO criteria. EndNote X9 reference manager software (Clarivate Analytics, Philadelphia, PA, USA) facilitated this process. Two independent authors (R.G.T. and Zs.T.) individually screened publications for title, abstract, and full text. To ensure the reliability of the selection process, Cohen’s kappa was calculated after both title and abstract selection, and again after full-text selection. This statistical measure quantified the inter-rater agreement beyond chance, adhering to Cochrane’s rigorous standards for conducting systematic reviews and meta-analyses.

### 2.5. Selection Protocol

#### Clinical Questions

The primary question guiding our review was: what is the most effective treatment for MBO in gynaecological malignancies?

### 2.6. Title and Abstract Selection

Both randomised and non-randomised studies were included, provided they involved adult women with MBO who underwent any kind of treatment. Studies or publications without original research data, such as reviews, letters, commentaries, and protocols, were excluded.

### 2.7. Full-Text Selection

Studies were included if they used the same measurement units for outcomes. Studies not matching the PICO framework or with inappropriate values were excluded.

### 2.8. Data Collection Process

Two authors (R.G.T. and Zs.T.) independently extracted data into an Excel spreadsheet (Office 365, Microsoft 16.86, Redmond, WA, USA).

#### Data Items

We collected the following data from the eligible articles: first author, year of publication, study type, study design, demographic data, details of treatments received, and data on outcomes for statistical analysis. The relief of the bowel obstruction symptoms can be measured through various methods, including improvements in symptoms such as abdominal pain, nausea, vomiting, and constipation. A third reviewer (L.L.) resolved discrepancies. 

### 2.9. Assessment of Bias and Assessment of Grade

The aim of this review was to extract, analyse, and compare outcome reports, counting their frequency, to determine the outcomes most commonly used in the evaluation of malignant bowel obstruction. This review did not intend to draw conclusions about treatment effectiveness, nor about the research design of the included studies.

To ensure the reliability of the included studies, we followed the NHS Executive guidelines from the Reviews on Commissioning Cancer Services. These guidelines categorise evidence quality by study design, from randomised controlled trials (Grade I) to cross-sectional studies (Grade IV). We assessed each study’s design, methodological quality (including sample size and follow-up duration), and potential biases (such as selection, performance, and drop-out biases). Outcome measures’ appropriateness and consistency were also reviewed. Two authors (R.G.T. and Zs.T.) independently reviewed each study, with discrepancies resolved by a third reviewer (L.L.), ensuring the inclusion of only high-quality studies [25].

### 2.10. Calculation of Cohen’s Kappa

In accordance with Cochrane’s rigorous standards for conducting systematic reviews and meta-analyses, Cohen’s kappa was calculated to protocolize the selection process and guarantee its systematic and comprehensive nature. The first calculation was made after title and abstract selection, and the second after full-text selection. Cohen’s kappa (*κ*) is calculated using the following formula:

$$\kappa =\frac{p_{0}-p_{e}}{1-p_{e}}$$

where *P*_0_ is the observed agreement, which is the proportion of agreement between the two raters, and *P_e_* is the expected agreement, which is the proportion of agreement that would be expected by chance alone. The observed agreement is calculated by summing the counts of items where the raters agree (the diagonal elements) and dividing by the total number of items.

## 3. Results

### 3.1. Included Studies 

Our systematic search resulted 5731 records. After eliminating 865 duplicates, 4866 articles underwent screening, resulting in the exclusion of 4788 during title and abstract evaluation. Additionally, 32 articles were excluded during full-text assessment. Subsequently, four articles were excluded due to data unsuitability, leaving 34 articles selected for systematic review. Inter-reviewer agreement was assessed using Cohen’s kappa (k = 0.84 for the first step and k = 0.87 for the second step of selection), with any discrepancies resolved by a third reviewer (L.L.). The characteristics of the studies identified for the systematic review, as well as the patient characteristics of the studies included, are detailed in Table 1. 

In total, 2068 patients were included from 34 studies. The studies identified were mainly observational studies, but there were no restrictions in the type of studies included. The inclusion criteria included all studies reporting management of malignant bowel obstruction associated with gynaecological malignancy, with no year of publication limitations and no limitations in the type of treatment and type of management or follow-up.

### 3.2. Medical Management

The medical management of malignant bowel obstruction (MBO) remains a significant challenge in clinical practice, particularly due to the limited number of studies available on various treatment options. Among these, the use of diatrizoate meglumine (Gastrografin), somatostatin analogues (octreotide), and dexamethasone has shown promising results in alleviating symptoms and aiding in surgical decision-making. This section reviews the current evidence on these medical treatments, highlighting their efficacy and role in the comprehensive management of MBO.

#### 3.2.1. Diatrizoate Meglumine 

A limited number of comparable studies are available on the use of Gastrografin in patients with malignant bowel obstruction. Heng’s retrospective analysis confirmed the efficacy of diatrizoate meglumine, with 84% of occlusions resolving after administration and 75% of these cases improving within the first 24 h. Notably, no significant complications were reported. The true value of diatrizoate meglumine lies in its ability to help determine the optimal timing for surgery. This is crucial, because conservative treatment success rates are low in cases of complete bowel obstruction, necessitating timely surgical intervention. Diatrizoate meglumine’s role is highly important in both symptom relief and the timing and decision-making of surgical interventions for patients with MBO [52]. 

#### 3.2.2. Somatostatin Analogues 

Octreotide emerged as the predominant medication investigated for managing MBO, with eight studies exploring its efficacy. The majority of patients in these studies were diagnosed with ovarian cancer, and no restrictions were observed based on the type of bowel involvement. Symptom resolution varied across studies, occurring within a timeframe spanning from 24 h to 4 days, with doses ranging from 100 μg/day to 0.9 mg/day. Two studies examined the use of octreotide in single doses [23,32]. Additionally, two randomised controlled trials (RCTs) included in the analysis revealed octreotide’s significant efficacy in symptom relief within 24 h compared to butylscopolamine [37,58].

In a comprehensive evaluation of the long-acting form of octreotide (LAR) in patients with recurrent ovarian cancer, Matulonis et al. administered 30 mg depot injections on Day 1 alongside subcutaneous octreotide for 2 weeks, providing sustained relief from bowel dysfunction. This approach demonstrated both safety and utility, with three out of fifteen patients experiencing a major reduction in malignant bowel obstruction (MBO) symptoms and two showing a minor response, while no significant toxicities related to octreotide or LAR were reported. Remarkably, some patients remained on LAR depot for over 9 months, suggesting its potential for long-term symptom management [40]. Similarly, Watari et al. investigated octreotide’s efficacy in controlling vomiting in patients with advanced gynaecologic cancer and inoperable gastrointestinal obstruction. Octreotide, administered via continuous infusion for two weeks, exhibited a high rate of vomiting control, with an overall response rate of 81.8%. Particularly noteworthy was its effectiveness in patients without nasogastric tubes, with an overall response rate of 93.1%. Furthermore, the absence of major adverse events associated with octreotide underscores its safety profile and potential to enhance quality of life by obviating the need for nasogastric tube placement in this patient population [42].

Walter et al. prospectively evaluated “triple therapy” consisting of dexamethasone, metoclopramide, and octreotide in managing non-surgical MBO. Despite the small sample size of 17 patients, the therapy exhibited promising results, with complete resolution of nausea and improvement in other symptoms such as pain and constipation. Although adverse effects such as bradycardia were noted in two patients, there were no incidences of bowel perforation [57].

A study conducted by Daniele et al. suggests that a tailored medical protocol, particularly involving anti-secretory drugs like octreotide, remains the standard of care for frail patients or those with contraindications to surgery [48].

While complications such as bowel perforation and necrosis were noted in a minority of cases, overall octreotide presents as a valuable adjunct in the management of MBO.

#### 3.2.3. Dexamethasone 

The use of dexamethasone in managing MBO in patients with gynaecological cancer has demonstrated promising outcomes. Our analysis compromising three studies involving 163 patients exclusively diagnosed with ovarian cancer highlights its efficacy. The dosage ranged from 4 mg/day to 8 mg twice a day. The use of dexamethasone is limited specifically to cases of small bowel obstruction, whether administered intravenously or subcutaneously. Dexamethasone typically achieves resolution of bowel obstruction within 5 to 7 days. While adverse events such as unpleasant perianal sensations are noted in some cases, overall success rates are encouraging, ranging around 89%. These findings underscore dexamethasone’s role as a valuable therapeutic option in managing MBO in patients with gynaecological cancer, offering relief and potentially improving their quality of life [34,36,55]. A summary of the abovementioned data is presented in Table 2. 

### 3.3. Invasive Interventions

#### 3.3.1. Percutaneous Gastrostomy

Percutaneous gastrostomy is a procedure used to insert a tube through the abdominal wall into the stomach. This tube provides a direct means of feeding or gastric decompression for patients who are unable to take adequate nutrition orally or who need relief from symptoms such as vomiting and nausea due to impaired gastric motility. Percutaneous gastroscopy can be performed surgically, endoscopically, or with radiologic intervention. In the case of MBO, the less invasive method is preferable, as the other procedures are highly symptomatic.

Patients treated with this option are usually not eligible for operations because of their general condition or their abdominal status, e.g., there are occlusions on more sites of the small bowel. However, this method is technically feasible, with a low rate of intervention failure. 

Studies consistently report high technical success rates, often close to 100% [27,29,30,38,39]. The reduction in symptoms, particularly nausea and vomiting, is substantial, with many studies reporting symptom relief in nearly all patients [31]. Gastrostomy has positive effects on the quality of life of the patients [49]. Survival times post-procedure vary significantly, with median or mean survival ranging from as short as 17 days to as long as 74 days [31,38]. Despite its effectiveness in symptom relief, PEG procedures are associated with complications, including leakage, peritonitis, site infections, and, in some cases, more severe issues like sepsis and autodigestion of the abdominal wall [27,30]. Overall, PEG demonstrates substantial efficacy in palliation for MBO, though the risk of complications necessitates careful patient management and selection [31,44,50,56]. Side effects and success rates are described in Table 3.

Total parenteral nutrition is a consequence of this intervention, although the majority of patients will be able to take sips of beverages for comfort after the insertion of gastric tubing. 

#### 3.3.2. Stent Placement

Gynaecological malignancies, especially ovarian cancer, can cause obstruction of the large bowel. It would be evident to use intraluminal colonic stents to restore the lumen of the bowel. Milek et al. investigated a new stent developed by themselves, which proved the efficacy and feasibility of the application of large bowel stents. In total, 85% of all patients felt decompression of the obstructed gastrointestinal tract after the first stent implantation [51]. In one case two stents were implanted due to insufficient coverage of the stricture. Another study led by Jutzi et al. showed that they had a technical success rate of 75% on their sample of 32 patients, whilst the clinical success rate was 47%, and 37.5% of the subjects had a complication requiring intervention [45]. 

Taking all these studies into account, the results are contradictory. Although the intervention is feasible and offers a good option for treatment, there was a meaningful need for intervention because of the complications caused by the stents. There is also a limitation by the nature of the disease, as it spreads on the peritoneal surface and can cause obstructions at different levels, while stenting is rather ideal for localised pathologies.

#### 3.3.3. Surgical Interventions

Surgery is the treatment option to choose when conservative methods do not seem to be effective in 3–7 days [35]. Surgical interventions can be demanding for the patient, and sometimes a longer period is needed for recovery, mainly for those patients whose baseline condition is already impaired. Thus, the widespread application of surgical interventions is limited, although they offer a longer symptom-free period and might prolong survival of the patients, even under palliative circumstances. Even though one must not lose the main objective of the procedure: pain relief and comfort enhancement.

Surgical interventions can improve quality of life, as there is a higher chance for patients to tolerate solid intake or fluids, and there is less need for total parenteral nutrition, like in the case of gastrostomies [23]. Chi et al. found that return of symptoms and death are less likely to occur in patients who went through a surgical intervention than those who received an endoscopic solution for their symptoms [41]. Despite all the negative effects, the reason to support surgical interventions in palliative cases is the fact that all the studies which we found in the literature proved a longer survival in this group of patients. 

It is still controversial who are the patients who can benefit from surgeries and what are the indicators of worse outcomes. Many studies examined this aspect and found correlations between survival and prognostic factors such as low albumin or elevated blood urea nitrogen or alkaline phosphatase or clinical factors like age, radiotherapy, ascites, carcinomatosis, multiple obstructions, or a palpable mass [26]. On the other hand, there are studies which do not support this [23]. 

In some very selected cases disseminated tumour spread results in a very complex surgery. Foutopoulou et al. found it feasible to perform these operations even if they resulted in short-bowel syndrome and consecutive total parenteral nutrition; however, they advised to treat these patients conservatively, as these operations can cause severe morbidity and a reduction in quality of life [43]. A later study conducted in the same centres by Armbrust et al. suggested that even these kinds of surgeries can extend therapeutic opportunities in highly selected patients [59]. 

It is essential to determine who are the ones who can benefit from surgeries and whose operation is feasible to avoid surgical failure and inappropriate interventions. Lodoli et al. suggest some features that could predict failure. According to them, surgery on a proximal occlusion has a higher chance of ineffectiveness due to shortening of the already anatomically short jejunal mesentery. Interestingly, they found that bowel dilatation without a real obstruction can result in surgical failure as well. Their explanation was decreased motility of the bowels because of widespread peritoneal infiltration that prevented surgeons from creating ostomies [54]. 

Previous studies suggest that age, disease extent, and nutritional status are important factors as well, from which they have created a risk stratification tool [28]. According to these and their own data, Perri et al. suggest a scoring system in which they take albumin level, presence of ascites more than 2 L, age, and non-ovarian tumour origin into account [46]. Another group of investigators found factors like ECOG status, platinum sensitivity, ascites  < 500 mL, type of stoma, and number of anastomoses to be the factors influencing results, emphasizing the importance of pre-operative frailty assessment [59]. Although, according to Daniele et al., cachexia, low performance status, and poor nutritional status emerged as significant predictors of worse survival, irrespective of the chosen treatment modality [48]. 

During the treatment of MBO it is crucial to have a reliable triage system to shorten the length of hospitalization and to avoid unnecessary surgical interventions in order to provide the highest quality of life possible. As MBO occurs subacutely, it is possible to start its treatment in an outpatient setting. Lee at al. developed an MBO programme which proved to achieve all its goals, as the rate of surgical interventions, frequency of recurrent episodes of MBO, and length of hospital stay were lower in the intervention group, and the possible chance of continuation of oncologic care was higher [53].

The studies included in the review were graded to monitor their quality according to the criteria set out by the NHS Executive in their Reviews on Commissioning Cancer Services. This grading ensured that only the strongest evidence was considered (Table 4). 

The basic characteristics of the studies included are summarized and detailed in Table 1, providing an overview of the patient demographics and study designs.

All the methods discussed in the manuscript have been summarised in Table 5 according to their advantages and disadvantages, providing a clear comparison for readers.

## 4. Discussion

Despite all the efforts and all the management options, malignant bowel obstruction remains a potentially lethal condition, with poor survival expectations [60,61]. Inadequately chosen therapeutic interventions may result in long-term hospitalisation or severe side effects [62,63]. However, under palliative circumstances the main aims are symptom and pain management and enhancement of quality of life, which should be reflected in the patient–physician communication as well [62,63]. Nevertheless, healthcare providers should seek for long-lasting therapeutic options, often using an invasive method, in order to provide the best quality of life achievable. Decisions about invasive measures must be made with the active involvement of the patient and the family of the patient. This is highlighted by the fact that personal factors, spiritual beliefs, and psychological and psycho-social factors play an important role in this process [64]. 

The active involvement of patients and their families is crucial, considering the frequency and severity of complications of potential surgical interventions. In order to decrease complications, numerous attempts have been made to determinate the factors influencing surgical outcomes; however, their results have often been inconclusive. Factors that appear to be reliable in evaluating potential risks include blood albumin levels, the presence of ascites, the complexity of the surgery (e.g., stoma placement, residual bowel length, number of bowel resections), general performance status, and frailty [28,46,65]. Furthermore, some authors suggest that patients’ life expectancy must be taken into consideration as well [19].

However, surgical solutions should be prioritised, as surgical interventions provide lower recurrence rate of obstructive episodes, longer symptom-free survival, and longer overall survival [35,41,48,66,67] than less demanding procedures. This statement is supported by a review of 868 patients carried out by Furnes et al. [68]. According to them, surgery was successful in relieving obstructive symptoms and provided the possibility of diet reintroduction and earlier discharge. Nevertheless, some authors suggest that longer survival rates may be attributed to the possible reintroduction of chemotherapy [69]. However, chemotherapy alone has not proved to be effective in the restoration of bowel function in heavily pretreated patients [70]. 

When a decision on surgery is made, interventions must be accomplished [71] in a moderate manner, keeping in mind the intention of the procedure. The chosen type of surgery depends on the location and the possible multiplicity of the obstruction, but always must be tailored to each single situation. Under these circumstances cytoreduction is no longer the goal of the operation, and symptom management procedures (e.g., bowel resections, bypasses, ostomies) should be carried out instead. 

In case of inoperability, the treatments of choice are less invasive measures: stent placement or percutaneous gastrostomy. Stents are recommended mainly in cases of large bowel or duodenal obstructions, when they are present solitarily [45]. The application of stents promises a high success rate for implantation, with a relatively low chance for further surgical intervention and prolonged survival [72,73]. 

If there are multiple sites of obstruction, gastrostomy seems to be the better solution for management. Gastrostomies are advised even in very vulnerable patients, as this procedure proved to be feasible in numerous studies and provided good symptom control, equally if they were performed radiologically or endoscopically [27,29,30,31,33,38,39,44,49,50,56]. Both gastrostomies and jejunostomies result in short bowel syndrome, leading to the necessity of total parenteral nutrition; thus, the involvement of nutritional specialists is inevitable in the management of these patients [74].

Until a decision on surgical intervention is made, administration of several drugs is needed in order to relieve the patient’s symptoms.

Some authors suggest that therapy should start with conservative management of MBO, as spontaneous or treatment-evoked resolution can happen in a great proportion of patients. However, conservative treatment might have a bridge function as well until the surgical intervention, as it offers the possibility of optimising the patient for intervention. 

Initial, symptomatic care should have three major directions, as follows: pain management, control of vomiting, and control of basic homeostatic parameters.

Supportive care must cover the restoration of basic homeostatic parameters. During pathophysiological changes, increased secretion of intestinal fluids, extravasation as a response to inflammatory reactions, and consequential emesis, significant electrolyte imbalance develops [75]. 

In the majority of the cases pain management cannot be carried out without the use of opioids. In this setting bowel movement inhibition plays an adjuvant role, as it reduces cramping. Following resolution of a malignant bowel obstruction (MBO) episode, it is essential to minimise the long-term use of opioids to avoid potential side effects and dependency. Pain management should be guided by the WHO analgesic ladder, which recommends a stepwise approach to pain relief. This involves starting with non-steroidal anti-inflammatory drugs (NSAIDs) and adjuvants, such as antidepressants or anticonvulsants, to address pain and enhance analgesic effects. If these medications prove insufficient, clinicians should then consider the use of minor opioids. Only if these measures fail to provide adequate pain relief should major opioids be introduced [76]. 

In a great part of cases bowel function can be restored using diatrizoate meglumine, which is used in the diagnostics of MBO routinely. Our search showed 84% effectivity in the restoration of the bowel function. A meta-analysis from Branco et al. [77] confirms this, as it concludes from 14 studies that, if the contrast material does not appear in the large bowel in 24 h, surgery is inevitable in 99% of cases. Parallelly, Galardi et al. found that patients who underwent a small bowel diatrizoate meglumine follow-through test were operated on earlier than those who did not [78].

In our systematic review we found that, in the management of vomiting, somatostatin analogues showed promising results in the management of MBO, reducing the main pathogenic event, which is the accumulation of bowel content. Somatostatin analogues decrease bowel motility and bowel and pancreatic secretion [18]. Therapeutic efficacy regarding symptom control proved to be at least 82%, and in most of the studies 100% symptom control was achieved in less than 4 days of application, without major complications. The preferred drug was mainly octreotide in a 0.3 mg/day dosage.

In the management of MBOs, corticosteroids were widely investigated according to their anti-inflammatory and anti-secretory attributes, reducing the amount of intraluminal contents and wall oedema by promoting water and salt absorption [19]. Among these substances the use of dexamethasone was the most common, showing a significant response in MBO, as complete response rate was achieved in at least half of the patients.

The most promising conservative treatment for MBO might be the combination of dexamethasone, lanreotide, and metoclopramide [79]; in this manner combined anti-inflammatory, secretion-reducing, and motility-restoring action can be achieved.

There is substantial evidence in the literature demonstrating that established palliative care programs significantly improve quality of life for patients. The integration of palliative care into general oncologic treatment should be implemented as early as possible to maximise benefits [80,81]. Oncologic patients having intra-abdominal disseminated cancer are at high risk of developing bowel obstruction. For this group of patients, it is important to provide dietary interventions and offer laxatives preventing constipation, which can exacerbate obstruction [47]. Assessing the chance of MBO, as early detection is shown to improve outcomes of necessary interventions and well-established local management protocols, can decrease hospital stay and improve quality of life for patients [53]. 

Besides clinical symptoms and physical examination, radiologic methods play a crucial role in setting the diagnosis of MBO. CT scan can have a higher value in further therapy management than conventional X-ray, considering its capability to determine the location and possible multiplicity of the obstruction. Contrast enhancement is widely used to determine obstructions, whether those are complete or not. Besides, in a remarkable part of cases the use of Gastrografin can resolve symptoms; nevertheless, its predictive value is the most important [52].

Parallel with diagnostics, clinicians must actively engage with the patients’ symptoms and start supportive care on pain management and electrolyte restoration.

Another direction of supportive care is to relieve nausea and vomiting, which can be rapidly decreased with the placement of a nasogastric tube (NGT). The use of nasogastric tubes belongs to basic care in cases of small bowel occlusion. The use of nasogastric tubing is fundamental in the care of patients with small bowel occlusion, primarily for symptom relief, but also helps to reduce distension of the stomach and small bowel. However, limited data support its routine use. In 2014 Paradis et al. [82] conducted a systematic search on data related to this topic, from 1966 to 2014. They found only one paper with relevant data, but this was not strong enough to make it into evidence [83]. According to this retrospective study conducted by Fonseca et al. [37], a significant association was found between NGT placement and the development of pneumonia and respiratory failure. Patients with NGTs had longer resolution times and extended hospital stays. Although NGTs can quickly relieve symptoms, their prolonged use can disturb patients and affect their quality of life. Nasogastric tubes are quite uncomfortable, and long-term use can provoke epistaxis, necrosis on the nasal wings, laryngeal disorders, regurgitation, and aspiration pneumonia, thus increasing the length of hospital stay and decreasing the most important value in the palliative setting, quality of life. These results highlight the need for selective NGT use, considering risks and benefits individually. However, the use of NGTs remains the standard of care in the acute phase. 

The most promising conservative treatment of MBO seems to be the combination of dexamethasone, octreotide, and metoclopramide.

**Figure 2:**
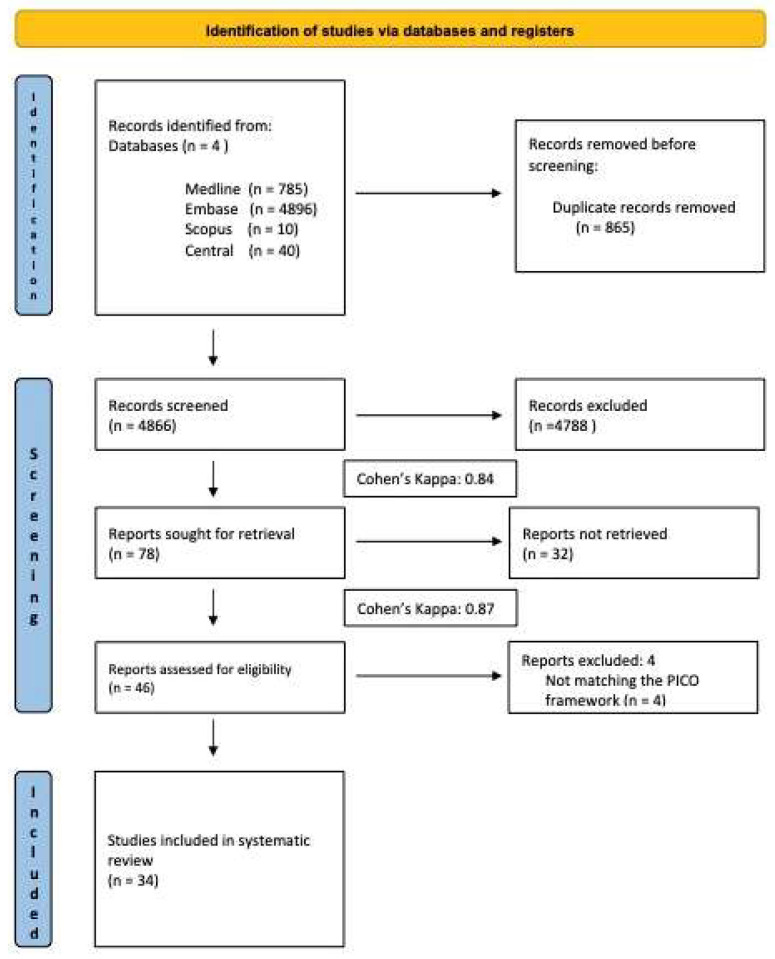
PRISMA flow diagram of the screening and selection process.

**Figure 3:**
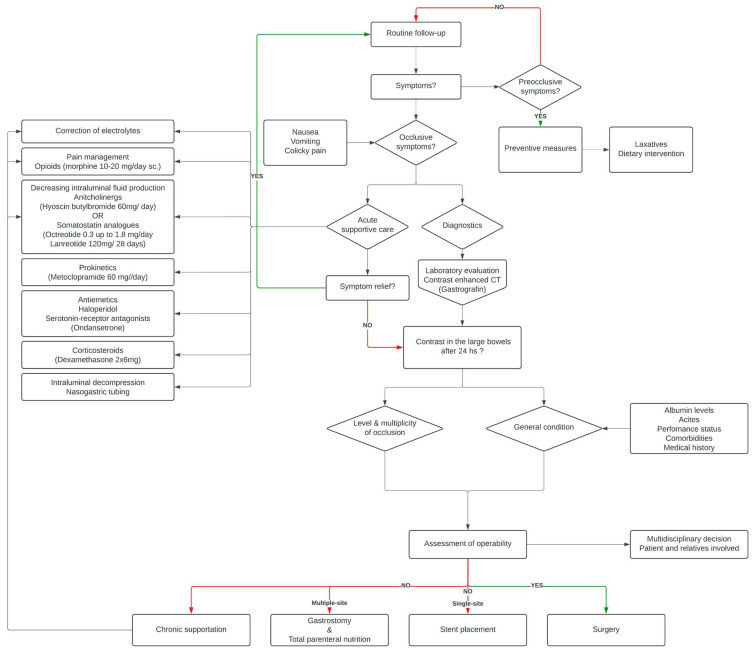
Therapeutic algorithm of MBO.

Synthesising all the data found, we tried to provide a treatment algorithm which can be a useful guide in the palliative care of patients with gynaecologic cancer suffering from malignant bowel obstruction (Figure 2). For a summary of the therapeutic algorithm see Figure 3.

Current society guidelines suggest that treatment algorithms must be managed individually, as evidence is lacking on treatment modalities, thus encouraging further investigations on this topic [17,64]. Review of the literature reveals that decision-making in the management of MBO is complex and not straightforward. Treatment plans must be personalised and involve a multidisciplinary team, considering all aspects of the patient’s condition. It is crucial to acknowledge that patients’ perspectives on their treatment may differ significantly from those of healthcare providers, necessitating a holistic approach in care [84]. 

To facilitate easier decision making, the development of risk-stratifying algorithms is necessary to identify patients who would benefit from surgery [85]; on the other hand, robust prospective trials are needed in this field to collect data on specific treatment modalities to be capable of creating universal guidance for the management of this patient population.

## 5. Conclusions

The optimal management of malignant bowel obstruction remains controversial. This review aimed to summarise the current literature to provide guidance on the management of this condition.

### 5.1. Implications for Practice

Given the limited availability of strong evidence, it is challenging to establish a single therapeutic approach for patients with malignant bowel obstruction. Our recommendation represents one perspective and highlights the need for individualised treatment strategies. Due to the absence of definitive guidelines, healthcare providers must tailor treatment plans to each patient’s specific circumstances, acknowledging that the objectives of treatment may differ between patients. The development of holistic, patient-centred management pathways is crucial.

### 5.2. Implications for Future Research

The objective of this study was to synthesise the latest data on malignant bowel obstructions in gynaecological cancers. Our systematic review underscores the lack of high-quality evidence, with most studies being retrospective, and the few prospective studies involving small patient cohorts. Data heterogeneity, originating from differences in patient populations, data collection methods, local management practices, and treatment intentions, further complicates comparability.

Future investigations should prioritise prospective data collection through multicentre international collaborations to generate robust evidence and address outstanding questions in the management of malignant bowel obstruction. 

## Figures and Tables

**Table 1:** Basic characteristics of the studies.

Author, Year, Grade	Population, Study Design, Duration of Study, Survival	Intervention	Outcome Measures	Notes/Side Effects
Castaldo et al. [26]; 1981	419 patients with ovarian cc. (between 1968 and 1977); retrospective study; group 1—mean survival was 16 months; group 2—mean survival was 18 months	group 1—intestinal surgery during their initial laparotomy; group 2—intestinal surgery during re-exploration, no symptoms; group 3—intestinal surgery during re-exploration, symptomatic	group 1—pts discharged within 18 days due to infrequent complications; group 2—infrequent complications but major when occurred; group 3—major complications	postoperative death; wound infection wound dehiscence; recurrent SBO; sepsis; enterocutaneous fistula; pulmonary embolus; GI bleeding
Malone et al. [27]; 1986	10 patients with ovarian cc; retrospective study; between November 1984 and August 1985; mean survival was 35 days	percutaneous gastrostomy	symptom reduction—10/10 (100%); technical success rate—10/10(100%)	1 leakage around tube, autodigestion of abdominal wall 1, pain 36 h 1, pyrexial 24 h 10/10 (100%)
Larson et al. [28]; 1989	33 patients with intestinal obstruction due to ovarian cc. (between 1980 and 1987); retrospective study; median survival time: 92 days without surgery and 102 days with surgery	surgical intervention	survival time significantly related to the prognostic index	N/A
Lee et al. [29]; 1991	12 patients with gynaecological cancer: 10 ovarian, 1 endocervical, 1 endometrial; retrospective study; duration of study—N/A; OS—N/A	interventional radiology	symptom reduction—12/12 (100%); technical success rate—12/12(100%)	1 peritonitis, 3 leakage
Cunningham et al. [30]; 1995	20 patients with gynaecological cancer: 10 ovarian, 6 endometrial, 3 cervical, 1 peritoneal; retrospective study; between July 1989 and June 1993; mean OS was 70 days	interventional radiology	symptom reduction—18/20; technical success rate—20/20 (100%)	1 sepsis, 2 leakage
Cannizzaro et al. [31], 1995	22 patients—14 ovarian, 5 endometrial, 3 colon cc.; prospective study; duration of study was 1 year; mean OS was 74 days (13–272)	endoscopy	symptom reduction—21/21 (100%); technical success rate—21/22 (95.5%)	1 spontaneous dislodgement, 1 persistent bloating, 1 mild site infection
Mangili et al. [32]; 1996	13 patients with gastrointestinal obstruction due to advanced ovarian cancer from January 1992 to May 1994; clinical trial; mean survival from discharge was 15 days (8/13 pts were discharged from the hospital); mean survival from the diagnosis of MBO was 27.1 days	8 pts—nasogastric drainage and 6 received parenteral nutrition/ hydration; octreotide—a starting dose of 0.3 up to 0.6 mg (mean 0.44 mg) a day by subcutaneous bolus or continuous infusion	complete relief of symptoms was achieved within 3.07 days (range 1 ± 6 days); vomiting stopped within 2 ± 3 days of starting treatment in most patients; in 8 pts with nasogastric tube, drainage decreased from 2000 to under 100 mL/day after the start of octreotide treatment	no side effects
Campagnutta et al. [33]; 1996	34 patients with gynaecological cancer: ovarian cc: 29 patients, endometrial cc: 2, uterine sarcoma in 2, and cervical carcinoma in 1; prospective study, not feasible for surgery	34 endoscopy PEG	27/32 (84.4%) symptomatic relief	4 patients: nausea, vomiting
Hardy et al. [34], 1998	patients with MBO due to ovarian cc.; trial 1: 25 pts; trial 2: 14 pts; combined: 39 pts; double-blind, placebo-controlled cross-over study; trial 1: 36-month period; trial 2: 24-month period; median overall survival (diagnosis to death) was 19 months	placebo (normal saline) or dexamethasone 4 mg intravenously (iv), every 6 h for five days	resolution of the bowel obstruction at day 5; response rate: trial 1: 15/22; trial 2: 6/13; combined: 21/35 (60%)	unpleasant perianal sensation
Gadducci et al. [35]; 1998	67 patients with epithelial ovarian cancer (between 1989 and 1997), 50.7% developed intestinal obstruction during the study; retrospective study; between 1989 and 1997; median survival was 23 months	22 patients: surgical interventions: - gastrostomy; jejunostomy; ileostomy; partial gastric resection; ileal resection; right or left colon resection; Hartman procedure; Sigmoid colostomy; transverse colostomy; ureter resection; ileo-ileal by-pass; 12 patients conservative therapy	from the 22 pts, 10 underwent further chemotherapy: died after a median interval of 275 days; the other 12 pts did not receive chemotherapy: died after a median interval of 45 days; 2 pts underwent further surgery for obstruction: died within 30 days	cardiovascular complications, bowel perforation, DIC, hematemesis, AML, cachexia
Philip et al. [36]; 1999	33 patients with MBO due to gynaecological cc. (mostly ovarian cc.); prospective cohort study; between 30 January 1994 and 30 January 1995; mean survival of the responding pts was 39 days	dexamethasone: 8 mg/day iv/sc 8 mg/day divided doses	9 pts (69%) had a response—decreased pain, nausea, and vomiting and improved oral intake (31 days)	patient 11: reduced dose because of mild proximal myopathy affecting the lower limbs
Mercadante et al. [37]; 2000	18 patient with inoperable bowel obstruction due to ovarian, vulva, rectum, pancreas, breast, stomach, liver, small bowel cc.; randomised controlled trial (RCT); OS—N/A; duration of study—N/A	octreotide (OCT) 0.3 mg daily vs. hyoscine butylbromide (HB) 60 mg daily	symptom relief within 24 h—OCT > HB	increased fluid intake correlated with less nausea
Brooksbank et al. [38]; 2002	51 patients—16 ovarian; retrospective study; between 1989 and 1997; median OS was 17 days	46 endoscopy, 4 laparotomy, 1 interventional radiology	symptom reduction—47/51 (92%), technical success rate—endoscopy 46/ 48 (96%), total 51/51 (100%)	1 hematoma, 6 leakage
Pothuri et al. [39]; 2005	94 patients with ovarian cancer; retrospective study; between 1995 and 2002; median OS was 8 weeks (95% CI, 6–10)	92 endoscopy, 2 interventional radiology	symptom reduction—86/94 (91%), technical success rate—94/94 (100%)	1 peritonitis, 8 leakage, 3 site infection, 3 blockage, 2 catheter malfunction, 2 bleeding
Matulonis et al. [40]; 2005	15 patients with MBO due to recurrent ovarian cancer; clinical pilot study; between 2002 and 2004; mean survival was 226 days, median survival was 89 days	100 μg OCT subcutaneously, followed by 30 mg LAR intramuscularly	complete symptom relief within 3.07 days, vomiting stopped within 2–3 days	no significant toxicities
Mangili et al. [23]; 2005	47 patients with intestinal obstruction due to recurrent epithelial ovarian cancer; retrospective study; duration of study—N/A; mean survival from the diagnosis of MBO was 79 days	27 patients—surgery (21 intestinal procedures, 2 gastrostomy tubes, 4 pts inoperable), 20 patients received octreotide (mean dosage of 0.48 mg/day), from which 1 patient required nasogastric tube	octreotide—controlled vomiting in all cases (except 1: NGT), complete symptom relief within 3 days; Surgery—16 of 21 pts (76%) tolerated low-residue diet	18% surgical correction not possible (mesentery infiltration); 22% complications: wound infection, dehiscence, fistula; oct-1 patient—fistula
Chi et al. [41]; 2009	26 patient with MBO due to ovarian cc.; prospective study; between July 2002 to July 2003; survival time: operative procedure: 191 days, endoscopic procedure: 78 days	PEG, colonic stent, intestinal bypass, ileostomy, colostomy	76% symptom relief	3.8% death, 11.5 % major complications
Watari et al. [42]; 2012	22 patients with MBO due to cc.; Endometrial or cervical cc.: 6 pts, ovarian cc.: 12 pts, peritoneal cc.: 3 pts, endometrial-ovarian cc.: 1 pt; prospective study; between 2006 and 2009; OS—N/A	300 μg/d OCT subcutaneously or intravenously as a continuous injection for 7 days + for another 7 days	15 patients (68.2%) had a response of CC, and 3 patients (13.6%) had a response of PC, with an overall response rate (CC/PC) of 81.8%	no side effects
Fotopoulou et al. [43]; 2013	37 patients with epithelial ovarian cc.; retrospective cohort study; between May 2003 and January 2012; median OS was 5.6 months;	surgical intervention, stent placement, conservative therapy	no significant differences in survival	any major complications 19 (51%): sepsis 1, pulmonary embolism 2, peritonitis 4, pleural effusion 3, relaparotomy 12, anastomotic insufficiency 5, abscess, secondary wound healing, postoperative bleeding 2, intestinal perforation 1, rupture of abdominal wall closure 1; peritonitis 100% short small bowel syndrome
Rath et al. [44]; 2013	53 patients with ovarian cc.; retrospective study; between 1/2002 and 12/2010; median OS was 46 days (2–736)	33 surgical, 13 interventional radiology, 6 endoscopy	symptom reduction—49/53 (93%), technical success rate—53/53 (100%)	9 blockage, 4 leakage, 5 site infection
Jutzi et al. [45]; 2014	32 patient with LBO and gynaecological malignancies (ovarian cc. 75%, uterine cc. 18,8%); retrospective cohort study; between January 2006 and February 2013; median survival time for all patients was 4.1 months	colorectal stent placement	clinical success 47%	complication rate = 42%, 12 stent -related complications in 10 pts:—obstruction, stent migration, bowel perforation, rectal bleeding, rectovaginal fistula, diarrhoea
Perri et al. [46]; 2014	62 patients with gastrointestinal obstruction due to gynaecological (47) malignancies (ovarian (69.1%), primary-peritoneal (8.8%), cervical (11.8%), or uterine (10.3%)); retrospective study; between October. 2004 and January 2013; median postoperative survival was 106 days	colostomy (26.5%), ileostomy (39.7%), colonic stent (1.5%), gastrostomy (7.3%), gastroenterostomy (5.9%), bypass/resection and anastomosis (19.1%)	18 pts died prior to discharge within 3–81 days; bypass/resection and anastomosis: improved survival	5 sepsis, 6 leak from anastomosis, 2 necrotizing fasciitis
Peng et al. [47]; 2015	97 patients with MBO due to advanced ovarian cancer; randomised controlled trial (RCT); between January 2010 and December 2013; OS—N/A	octreotide (OCT) 0.3 mg/day vs. scopolamine butylbromide (SB) 60 mg/day	symptom relief within 24 h—OCT > SB	N/A
Daniele et al. [48]; 2015	40 patients with MBO due to ovarian cancer; retrospective study; between October 2008 and January 2014; medical treatment group: median survival from MBO was 5,7 months; surgical treatment group: median survival from MBO was 13.6 months;	18 pts—medical treatment: morphine sulfate 60 mg, haloperidol 1.5 mg, OCT 0.3 mg, dexamethason 8 mg /d; 22 pts—surgery	symptom relief within 4 days	no side effects
Zucchi et al. [49]; 2016	158 patients—96 ovarian, 13 colon, 8 endometrial, 41 other cc.; prospective study; between 2002 and 2012; Median OS was 57 days (4–472)	endoscopy	symptom reduction—110/142 (77%) complete, 12/142 (8%) controlled vomiting, technical success rate—142/158 (90%)	3 dislodged, 20 site infection, 12 obstruction, 2 leakage, 3 bleeding, 1 catheter failure
Dittrich et al. [50]; 2017	76 patients—ovarian 24 (32%), colorectal 13 (17%), pancreatic 12 (16%), small intestine 5 (7%), gallbladder/biliary tract 5 (7%), gastric 4 (5%), breast 3 (4%), CUP 3 (4%), other 6 (8%); Retrospective study	endoscopy—PEG	significant decrease of vomiting (*p* < 0.001)	112 complications in 56 patients: stomal leakage (18/75 patients), mild wound pain (17/75), and tube occlusion (13/75) occurred most frequently
Miłek et al. [51]; 2017	13 patients with left half colon obstruction due to an inoperable metastatic ovarian cc.; prospective study; 2012–2014	colorectal stent placement	successful decompression in 11 pts (85%)	1 patient with stent migration (7.7% in 24 h), 1 outgrowth of the neoplasm beyond the upper edge of the stent and subsequent stricture of the intestine’s lumen (4 months)
Heng et al. [52]; 2018	71 patients (47 women): 24 (33.8%) with ovarian or primary peritoneal neoplasms, 14 (19.7%) bowel, 8 (11.2%) upper gastrointestinal, 5 (7%) pancreatic, 6 (8.5%) intra-abdominal neoplasms, 2 (2.8%) other neoplasms with intra-abdominal/peritoneal metastases, 12 (16.9%) other neoplasms without intra-abdominal/peritoneal metastases; intestinal obstruction in 42 (59.2%) patients; retrospective study; between January 2013 and October 2015 (approximately 34 months), OS—N/A	50 mL Gastrografin—repeated small doses over several days	resolved occlusion in 84% after administration, 75% of these cases improving within the first 24 h	10 patients (14%)—diarrhoea
Lee et al. [53]; 2019	169 patients with MBO due to advanced gynaecological malignancies; retrospective cohort study; baseline program between 2014 and 2016, MBO program between 2016 and 2018; median OS: 141 days MBO program: 141 vs. baseline: 99	surgery, chemotherapy, total parenteral nutrition, and supportive care	shorter hospital length of stay in the MBO program group compared to the baseline group	N/A
Lodoli et al. [54]; 2021	76 patients with MBO due to gynaecological (67), GI (19), and other (12) malignancies; retrospective observational cross-sectional study; study time was 5 years (between 2014 and 2018); OS—N/A	colostomy 7.2%, ileostomy 62.3%, jejunostomy 30.4%, intestinal bypass, bowel resection, adhesiolysis	Surgery achieved 77.5% 68% p.o. diet, 61.2% NPT, 49% hospice, 51% home	21.4% complication, 9.2% major
Jones et al. [55]; 2022	91 patients with epithelial ovarian cancer, partial or complete bowel obstruction; retrospective cohort study; between January 2005 and December 2016; median survival from the diagnosis of MBO was 3.8 months	dexamethasone: median daily dose: 6–8 mg, twice daily; median total dose was between 26 and 40 mg	89% (137 admissions); 44.8%—adequate symptom resolution	N/A
Armbrust et al. [49]; 2022	87 patients with ovarian cc.; retrospective cohort study; between 2012 and 2017; mean OS was 7.8 months	5% colectomy or total colectomy, 46% small bowel resection, 12% primary anastomosis	ECOG status, platinum sensitivity, ascites < 500 mL, the type of stoma and the number of anastomosis influenced the results	42% TPN, 26% grade 3 complication, 13% secondary wound healing, 21% anastomotic leakages, transfusions (17%) or thromboembolic events, 30 d mortality—10% 30 d morbidity—74%
Cole et al. [56]; 2023	14 patients—8 gynaecologic, 3 colorectal, 1 bladder, 1 small bowel, 1 peritoneal serous; retrospective study; between November 2019 and July 2021; mean OS was 270 days	endoscopy	symptom reduction—100%, technical success rate—100%	N/A
Walter et al. [57]; 2024	17 patients (8 women) with MBO due to UG, GI, GYN, lung cancer; prospective study; between 21 October 2019 and 1 December 2021; overall median survival was 88.8 days; 6 months survival was 20%	“triple therapy”: dexamethasone 4 mg BID, metoclopramide 10 mg Q6 and octreotide 300 mcg TID	10 patients (66.7%)—deobstruction; resolution of the bowel obstruction or deobstruction was defined as, introduction of oral intake beyond sips of liquids with cessation of vomiting and or ability to remove nasogastric tube (NGT) or tolerance of clamped venting gastrostomy tube (GT), resumption of bowel movements	bradycardia in 2 pts, no incidence of bowel perforation

Abbreviations: NR: not reported, OS: overall survival, OC: ovarian cancer, EC: endometrial cancer, CC: cervical cancer, PC: peritoneal cancer, CRC: colorectal cancer, UC: uterine cancer, BC: bladder cancer, SB: small bowel, Gyn: gynaecologic malignancy, Pan.: pancreatic cancer.

**Table 2:** General characteristics of the articles regarding medical treatment.

Author	Study Type	Methods	OS (MD/MN)	Symptom Relief	Notes/Side Effects
Hardy et al. [34] (1998)	Double-blind, placebo-controlled, cross-over	Placebo or DEX 4 × 4 mg/day iv, for five days n = 39	570 days (MD)	CR: 60%	unpleasant perianal sensation
Philip et al. [36] (1999)	Prospective cohort	DEX: 8 mg/day iv/sc n = 33	39 days (MN)	OR: 69%	mild proximal myopathy affecting the lower limbs
Mercadante et al. [37] (2000)	Randomised controlled trial	OCT 0.3 m/day vs. HB 60 mg/day n = 18	N/A	CR in 24 h: OCT > HB	increased fluid intake correlated with less nausea
Mangili et al. [23] (2005)	Retrospective	OCT n = 20	79 days (MN)	CR: 95% in 3 days	one patient fistula
Matulonis et al. [40] (2005)	Prospective interventional cohort study	0.1 mg OCT sc, +30 mg LAR im n = 15	226 days (MN)	CR in 3.07 days	no significant toxicities
Watari et al. [42] (2012)	Prospective interventional cohort	OCT: 0.3 mg/ days sc/iv for 7 + 7 days n = 22	N/A	CR: 68.2% PR: 13.6%	no side effects
Daniele et al. [48] (2015)	Retrospective observational	MS 60 mg/day, HAL 1.5 mg/day, OCT 0.3 mg/day, DEX 8 mg/day; n = 18	171 days (MD)	CR 100% in 4 days	no side effects
Peng et al. [58] (2015)	Randomised controlled trial	OCT 0.3 mg/day vs. SB 60 mg/day n = 97	N/A	CR in 24 h: OCT > SB	N/A
Heng et al. [52] (2018)	Retrospective	50 mL Gastrografin—repeated small doses over several days n = 71	N/A	CR: 84%75% in 24 h	10 patients (14%)—diarrhoea
Jones et al. [55] (2022)	Retrospective cohort	DEX: 2 × 6–8 mg/day; n = 91	114 days (MD)	CR: 44.8%	N/A
Walter et al. [57] (2024)	Prospective interventional cohort	“triple therapy”: DEX: 2 × 4 mg/day, MCP: 4 × 10 mg/day, OCT: 2 × 0.3 mg/day n = 17	88.8 days (MD)	CR: 66.7%	bradycardia in two patients

Abbreviations: OCT: octreotide, SB: scopolamine butylbromide, HB: hyoscine butylbromide, LAR: lanreotide, MD: median, MN: mean, sc: subcutanenous, im: intramuscular, DEX: dexamethasone, MCP: metoclopramide, iv: intravenously, MS: morphine sulfate, HAL: haloperidol, CR: complete response, PR: partial response, OR: overall response.

**Table 3:** Studies evaluating gastrostomy outcomes in gynaecologic malignancies.

Author	Study Type	Method of Gastrostomy Formation	Number of Cases and Cancer Type	OS	Symptom Relief	Diet	Notes/Side Effects	Technical Success
Malone et al. [27] (1986)	Retrospective	Transsectional radiology	n = 10 OC:10	Mean: 35 days (26–56)	10/10 (100%)	NR	OA: 100% Fever: 10 Leakage: 1 Abdominal wall autodigestion: 1 Pain for 36 h: 1	10/10 (100%)
Lee et al. [29] (1991)	Retrospective	Interventional radiology	n = 12 OC: 10 CC: 1 EC: 1	NR	12/12 (100%)	NR	OA: 33% Peritonitis: 1 Leakage: 3	12/12 (100%)
Cannizzaro et al. [31] (1995)	Prospective	Endoscopy	n = 22 OC:14 EC: 5 CRC: 3	Mean 74 days (13–272)	21/21 (100%)	21/21 (100%)	OA: 14% Dislodgement: 1 Persistent bloating: 1 Mild site infection: 1	21/22 (95.5%)
Cunningham et al. [30] (1995)	Retrospective	Interventional radiology	n= 20 OC: 10 EC: 6 CC: 3 PC: 1	Mean 70 days (3–173)	18/20 (90%)	12/20 (100%)	OA: 15% Sepsis: 1 Leakage: 2	20/20 (100%)
Campagnutta et al. [33] (1996)	Prospective	Endoscopy	n = 34 OC: 29 EC: 2 UC: 2 CC: 1	Tube in place for median 74 days (5–210)	27/32 (84%)	27/32 (84%)	OA: 6% Mild site infections: 2	32/34 (94%)
Brooksbank et al. [38] (2002)	Retrospective	Endoscopy/ Laparotomy	n = 51 CRC: 27 OC: 16 Other: 8	Median 17 days (1–190)	47/51 (92%)	NR	OA: 14% Hematoma: 1 Leakage: 6	51/51 (100%)
Pothuri et al. [39] (2005)	Retrospective	Interventional radiology	n = 94 OC: 94	Median 8 weeks (95% CI, 6–10)	86/94 (91%)	89/92 (2 unknown) (97%)	OA: 20% Peritonitis: 1 Leakage: 8 Site infections: 3 Blockage: 3 Catheter malfunction: 2 Bleeding: 2	94/94 (100%)
Rath et al. [44] (2013)	Retrospective	Endoscopy	n = 53 OC: 53	Median 46 days (2–736)	49/53 (93%)	48/53 (91%)	OA: 34% Blockage: 9 Leakage: 4 Site infections: 5	53/53 (100%)
Zucchi et al. [49] (2016)	Prospective	Endoscopy	n = 158 OC: 96 CRC: 13 EC: 8 Other: 41	Median 57 days (4–472)	110/142 (77%) complete 12/142 (8%) vomiting controlled	110/142 (77%)	OA: 26% Dislodged: 3 Site infection: 20 Obstruction: 12 Leakage: 2 Bleeding: 3 Catheter failure: 1	142/158 (90%)
Dittrich et al. [50] (2017)	Retrospective	Endoscopy	n = 76 OC: 26 CRC: 13 Pan.: 12 Other: 25	Median 28 days (2–440)	96% (73/75) vomiting 81% (62/75) nausea	59/75 (79%)	OA: 53% Peritonitis: 3 Severe bleeding: 2 Repeated attempts: 7 Fever: 6 Leakage: 18 Wound infection: 9	68/76 (90%) primary 75/76 (99%) secondary
Cole et al. [56] 2022	Retrospective	Endoscopy	n = 14 Gyn: 8 CRC: 3 BC: 1 SB: 1 PC: 1	Mean 270 days	100%	NR	NR	100%

Abbreviations: NR: not reported, OS: overall survival, OC: ovarian cancer, EC: endometrial cancer, CC: cervical cancer, PC: peritoneal cancer, CRC: colorectal cancer, UC: uterine cancer, BC: bladder cancer, SB: small bowel, Gyn: gynaecologic malignancy, Pan.: pancreatic cancer.

**Table 4:** Grading of used literature.

Author, Year	Methods	Grade
Castaldo et al. [26]; 1981	retrospective observational	IIIA
Malone et al. [27]; 1986	retrospective observational	IIIA
Larson et al. [28]; 1989	retrospective observational	IIIA
Lee et al. [29]; 1991	retrospective observational	IIIA
Cunningham et al. [30]; 1995	retrospective observational	IIIA
Cannizzaro et al. [31], 1995	prospective single arm interventional study	IIA
Mangili et al. [32]; 1996	retrospective observational	IIB
Campagnutta et al. [33]; 1996	prospective single arm interventional study	IIB
Hardy et al. [34]; 1998	prospective placebo-controlled cross-over study	IIIA
Gadducci et al. [35]; 1998	retrospective observational	IIIA
Philip et al. [36]; 1999	prospective cohort	IIA
Mercadante et al. [37]; 2000	randomised controlled trial	IC
Brooksbank et al. [38]; 2002	retrospective observational	IIIA
Mangili et al. [23]; 2005	retrospective observational	IIIA
Matulonis et al. [40]; 2005	prospective single-arm interventional study	IIC
Pothuri et al. [39]; 2005	retrospective observational	IIIA
Chi et al. [41]; 2009	prospective study	IIA
Watari et al. [42]; 2012	prospective single-arm interventional study	IIA
Rath et al. [44]; 2013	retrospective observational	IIIA
Fotopoulou et al. [43]; 2013	retrospective observational	IIIA
Jutzi et al. [45]; 2014	retrospective observational	IIIA
Perri et al. [46]; 2014	retrospective observational	IIIA
Peng et al. [58]; 2015	randomised controlled trial	IB
Daniele et al. [48]; 2015	retrospective observational	IIIA
Zucchi et al. [49]; 2016	prospective single-arm interventional study	IIA
Dittrich et al. [50]; 2017	retrospective observational	IIIB
Miłek et al. [51]; 2017	prospective single-arm interventional study	IIB
Heng et al. [52]; 2018	retrospective observational	IIIA
Lodoli et al. [54]; 2021	retrospective observational	IIIA
Jones et al. [55]; 2022	retrospective observational	IIIA
Armbrust et al. [59]; 2022	retrospective observational	IIIA
Cole et al. [56]; 2023	retrospective observational	IIIA
Walter et al. [57]; 2024	prospective single-arm interventional study	IIA

Grading was performed according to the criteria set out by the NHS Executive in their Reviews on Commissioning Cancer Services.

**Table 5:** Treatment Strategies for Malignant Bowel Obstruction: Benefits and Drawbacks.

Treatment Method	Advantages	Disadvantages
Somatostatin analogues	− Effective in symptom relief within 24 h to 4 days − Can be used long-term with minimal toxicity − Reduces bowel and pancreatic secretion	− Requires continuous infusion or regular administration − May not be effective in all patients
Dexamethasone	− Effective in symptom relief within 5 to 7 days − High success rates (around 89%) − Useful in cases of small bowel obstruction	− Limited use in small bowel obstruction cases − Adverse events such as unpleasant perianal sensations in some patients
Diatrizoate meglumine	− Effective in restoring bowel function (84% success rate)	− If contrast material does not appear in the large bowel within 24 h, surgery is inevitable in 99% of cases
Percutaneous gastrostomy	− Feasible in very vulnerable patients − Good symptom control	− Leads to short bowel syndrome, requiring total parenteral nutrition − Not suitable for patients with multiple obstructions
Stent placement	− High success rate for implantation − Relatively low chance of requiring further surgical intervention − Prolonged survival in certain cases	− Recommended mainly for large bowel or duodenal obstructions − Less effective if multiple obstructions are present
Surgical interventions	− Provides definitive relief from obstruction − Prolonged symptom-free periods	− High morbidity and mortality rates − Not suitable for all patients, especially those with poor overall health

## Data Availability

The published article contains all generated and analyzed data from this series.
